# Stimulus effects of extremely low‐frequency electric field exposure on calcium oscillations in a human cortical spheroid

**DOI:** 10.1002/bem.22521

**Published:** 2024-08-25

**Authors:** Atsushi Saito, Takeo Shiina, Yoichi Sekiba

**Affiliations:** ^1^ Sustainable System Research Laboratory, Biology and Environmental Chemistry Division Central Research Institute of Electric Power Industry (CRIEPI) Abiko Japan; ^2^ Grid Innovation Research Laboratory, Electric Facility Technology Division Central Research Institute of Electric Power Industry (CRIEPI) Yokosuka Japan; ^3^ Power System Analysis Group, Denryoku Computing Center (DCC) Komae Japan

**Keywords:** electric field, human cortical spheroid, low‐frequency, nerve stimulation, neuronal activity

## Abstract

High‐intensity, low‐frequency (1 Hz to 100 kHz) electric and magnetic fields (EF and MF) cause electrical excitation of the nervous system via an induced EF (iEF) in living tissue. However, the biological properties and thresholds of stimulus effects on synchronized activity in a three‐dimensional (3D) neuronal network remain uncertain. In this study, we evaluated changes in neuronal network activity during extremely low‐frequency EF (ELF‐EF) exposure by measuring intracellular calcium ([Ca^2+^]_*i*_) oscillations, which reflect neuronal network activity. For ELF‐EF exposure experiments, we used a human cortical spheroid (hCS), a 3D‐cultured neuronal network generated from human induced pluripotent stem cell (hiPSC)‐derived cortical neurons. A 50 Hz sinusoidal ELF‐EF exposure modulated [Ca^2+^]_*i*_ oscillations with dependencies on exposure intensity and duration. Based on the experimental setup and results, the iEF distribution inside the hCS was estimated using high‐resolution numerical dosimetry. The numerical estimation revealed threshold values ranging between 255–510 V/m (peak) and 131–261 V/m (average). This indicates that thresholds of neuronal excitation in the hCS were equivalent to those of a thin nerve fiber.

## Highlights

- **Neuronal Network Assessment:** Using human cortical spheroids, we measured changes in neuronal network activity using [Ca^2+^]_*i*_ oscillations.
- **Electrical Stimulation Thresholds:** Thresholds for electrical stimulation were determined under exposure to 50 Hz sinusoidal wave extremely low‐frequency electric fields, revealing sensitivity patterns.
- **Quantified Exposure Levels:** Peak and average exposure levels were identified, providing quantifiable insights into the effects of induced electric field stimulation on neuronal network activity.

## 1 INTRODUCTION

Low‐frequency (1 Hz to 100 kHz) electric and magnetic fields (EF and MF) can stimulate nerves and muscles via induced electric fields (iEFs) in the human body (WHO, 2007). The stimulus effect on the nervous system is an established biological effect of time‐varying electromagnetic fields on the human body and serves as the biological basis for safety limits in the guidelines or standards for the protection of the human body established by the International Commission on Non‐Ionizing Radiation Protection (ICNIRP) (ICNIRP, 2010) and the electromagnetic field safety standards of the Institute of Electrical and Electronics Engineers (IEEE) (IEEE, 2019). However, the ICNIRP has issued a statement emphasizing the need to investigate the threshold of stimulus effects, such as neuronal network firing patterns and pain perception (ICNIRP, 2020). Comprehensive scientific evidence from human, animal, cellular, and numerical dosimetry and modeling studies is needed to address these issues.

Cellular studies using cultured neuronal networks facilitate the detection of neuronal tissue response in real‐time during high‐intensity EF or MF exposure, which is ethically difficult in human subjects. In addition, cultured neuronal networks allow for the detailed evaluation of thresholds and mechanisms from the cellular to the network level. In recent years, three‐dimensional (3D) neuronal networks derived from human induced pluripotent stem cells (hiPSCs) have been used to mimic the complexity of the human brain and some human neurodegenerative diseases (Centeno et al., 2018). In addition, the use of 3D neural networks, such as hiPSC‐derived neural spheroids and organoids, for EF or MF exposure experiments has been demonstrated (Consales et al., 2021; Meng et al., 2023; Saito, 2021; Ye et al., 2021). Therefore, the use of human neuronal tissue with a 3D structure for EF or MF exposure experiments is expected to support the mechanistic aspects of nerve stimulation in the human central nervous system. However, when using 3D neuronal culture aggregates, it is likely that the intensity, spatial distribution, and direction of the iEF within the neuronal tissue caused by the low‐frequency EF or MF may be more complexly distributed in the tissue than in conventional two‐dimensional (2D) cultures. Although previous cellular studies have estimated the distribution of iEFs on the surface of culture solutions using numerical dosimetry, few studies have examined the distribution of iEFs within cell aggregates. In addition, 3D neuronal networks have a higher density than 2D neuronal networks do, and the intrinsic patterns of neuronal activity may differ between them.

To investigate the threshold and difference of nerve stimulation caused by low frequency EF or MF exposure, the aim of this preliminary study was to evaluate the response of a 3D neuronal network to extremely low‐frequency EF (ELF‐EF) exposure and to assess the iEF intensity inside the 3D neuronal networks. Here, rhythmic and synchronized intracellular Ca^2+^ ion ([Ca^2+^]_*i*_) oscillations were observed during the development of hiPSC‐derived 3D neuronal networks, which were associated with electrical activity similar to that during human brain function (Boutin et al., 2022; Hörberg et al., 2022; Samarasinghe et al., 2021; Trujillo et al., 2019; Woodruff et al., 2020; Zourray et al., 2022). In addition, [Ca^2+^]_*i*_ oscillations can be observed using fluorescence imaging, which has the advantage of facilitating the real‐time detection of neuronal activity during EF or MF exposure without the influence of heat and electromagnetic noise (Saito, Terai, et al., 2018). Therefore, we produced human cortical spheroids (hCSs) derived from hiPSC‐induced neuronal progenitor cells to evaluate the stimulus effects of ELF‐EF exposure. Furthermore, to estimate the iEF distribution in hCSs, we performed a numerical calculation using a 3D model of hCS with a diameter of 1 mm and resolution of 6.25 µm. Based on these experiments, we estimated the threshold of stimulus effects on [Ca^2+^]_*i*_ oscillation in hCSs under ELF‐EF exposure.

## 2 MATERIALS AND METHODS

### 2.1 Cell culture

We used hiPSC‐derived neuronal progenitor cells (ReproNeuro; ReproCell) to generate the hCSs. According to the manufacturer, the induction of differentiation using ReproNeuro showed that 60%–80% of excitatory neurons harbored glutamatergic vesicle transporters, and 30%–50% of inhibitory neurons contained γ‐aminobutyric acid. In addition, differentiated cell populations were observed to contain 10%–30% dopaminergic neurons with tyrosine hydroxylase, and 10% cholinergic neurons harbored choline acetyltransferase. Differentiation of human neural progenitor cells (NPBs) into human cortical neurons was performed as previously described (Saito, 2021). ReproNeuro MQ medium (RMQ culture solution; ReproCell), a culture solution for the induction of hiPSC‐derived.

NPBs (hNPBs), was used to differentiate hCSs and prepared the day before seeding. On the day of seeding, hNPBs were removed from the liquid nitrogen container and thawed in a water bath, followed by the addition of 10 mL Dulbecco's modified Eagle's medium/F‐12 culture solution (11320033; Thermo Fisher Scientific) to prepare a cell suspension with a seeding density of 3 × 10^4^ cells/mL. The supernatant was removed via centrifugation (350*g*, 5 min), and 10 mL of the RMQ culture solution added to prepare the cell suspensions. The resulting cell suspension was seeded into each well of a 96‐well plate (MS‐9096 U; Sumitomo Bakelite) on a low‐adhesion substrate at a cell density of 3 × 10^4^ cells/well. The hNPBs were differentiated into cortical neurons by replacing the RMQ culture solution at 3, 7, and 14 days in culture. As long‐term culture is required for functional neuronal network formation in hCSs, we also used NPB medium based on the B‐27 Plus Neuronal Culture System (A3653401; Thermo Fisher Scientific) from Day 21. The medium contained 2% GlutaMAX supplement (35050061; Thermo Fisher Scientific) and 1% penicillin‐streptomycin (15140122; Thermo Fisher Scientific). The hCSs were cultured in NPB medium from the third week after differentiation. Thereafter, half of the medium was replaced with fresh NPB medium every 2–3 days. Using the method described above, we generated hCS cell clusters with a 3D structure through long‐term culturing in 96‐well plates for several months. In addition, to assess functional maturation of the neural network following long‐term culture, pharmacological responses were evaluated using D‐(‐)‐2‐amino‐5‐phosphonopentanoic acid (D‐AP5) (A8054; Sigma‐Aldrich), 6‐cyano‐7‐nitroquinoxaline‐2,3‐dione (CNQX) (C127; Sigma‐Aldrich), and 1(*S*),9(*R*)‐(‐)‐bicuculline methiodide (BMI) (14343; Sigma‐Aldrich) as inhibitors of excitatory or inhibitory neurons. Cell culture was performed in 5% CO_2_ at 37°C.

### 2.2 Extracellular recording

To evaluate neuronal network activity during hCS development, extracellular potential recordings were performed using a multi‐electrode array (MEA) recording system at each stage of the neuronal network development process. Extracellular potentials were recorded using a MED64 system (Alpha MED Scientific [Screen]). Electrodes (50 × 50 µm) were arranged in an 8 × 8 grid on the bottom of the MEA dish (MED‐R515A; Alpha MED Scientific [Screen]). To place the hCS on the electrodes, a silicon chamber (Alpha MED Scientific [Screen]), which comprised an annular hole with a diameter of 3 mm and height of 1 mm, was placed on the 8 × 8 electrodes. Before electrical recording, we positioned an hCS on the electrodes using this silicon chamber. The recorded signals were amplified by a factor of 10,000 using both preamplifiers and postamplifiers, and a bandpass filter applied from 100 Hz to 2 kHz. The electrical signals recorded by the MED64 system were converted to digital data at a sampling frequency of 25 kHz using an A/D converter and stored on a computer. Measurement data display and analysis were performed using dedicated software (Mobius; Alpha MED Scientific [Screen]) supplied with the MED64 system. The signal analysis detected spontaneous spike‐firing in the hCS, as well as the changes in firing frequency, at each developmental stage and pharmaceutical application.

### 2.3 Calcium imaging

To evaluate stimulus‐induced responses to ELF‐EF exposure and its effect on neuronal activity, intracellular calcium ([Ca^2+^]_*i*_) in the cell membrane after hCS excitation was assessed using fluorescent calcium imaging. The Quest Fluo‐8 AM cell‐permeable fluorescent indicator (AAT BioQuest) was used for calcium imaging. The indicator was introduced by adding 10 µM Fluo‐8 AM to the culture solution immediately before the start of the ELF‐EF exposure experiment, and allowed to act for at least 30 min in a CO_2_ incubator. For real‐time detection of [Ca^2+^]_*i*_ changes during ELF‐EF exposure, the exposure apparatus was combined with a fluorescence microscopy system. In this study, changes in fluorescence intensity (Δ*F*) associated with [Ca^2+^]_*i*_ oscillations were expressed as a fluorescence intensity ratio (Δ*F*/*F*) normalized to the background fluorescence intensity (*F*). The [Ca^2+^]_*i*_ bursts and spikes detected during EF‐ELF exposure were analyzed via a one‐sample *t*‐test. Data are expressed as the mean ± standard deviation, and statistically significant differences defined as *p < 0.05.

### 2.4 ELF‐EF exposure

**Figure 1:**
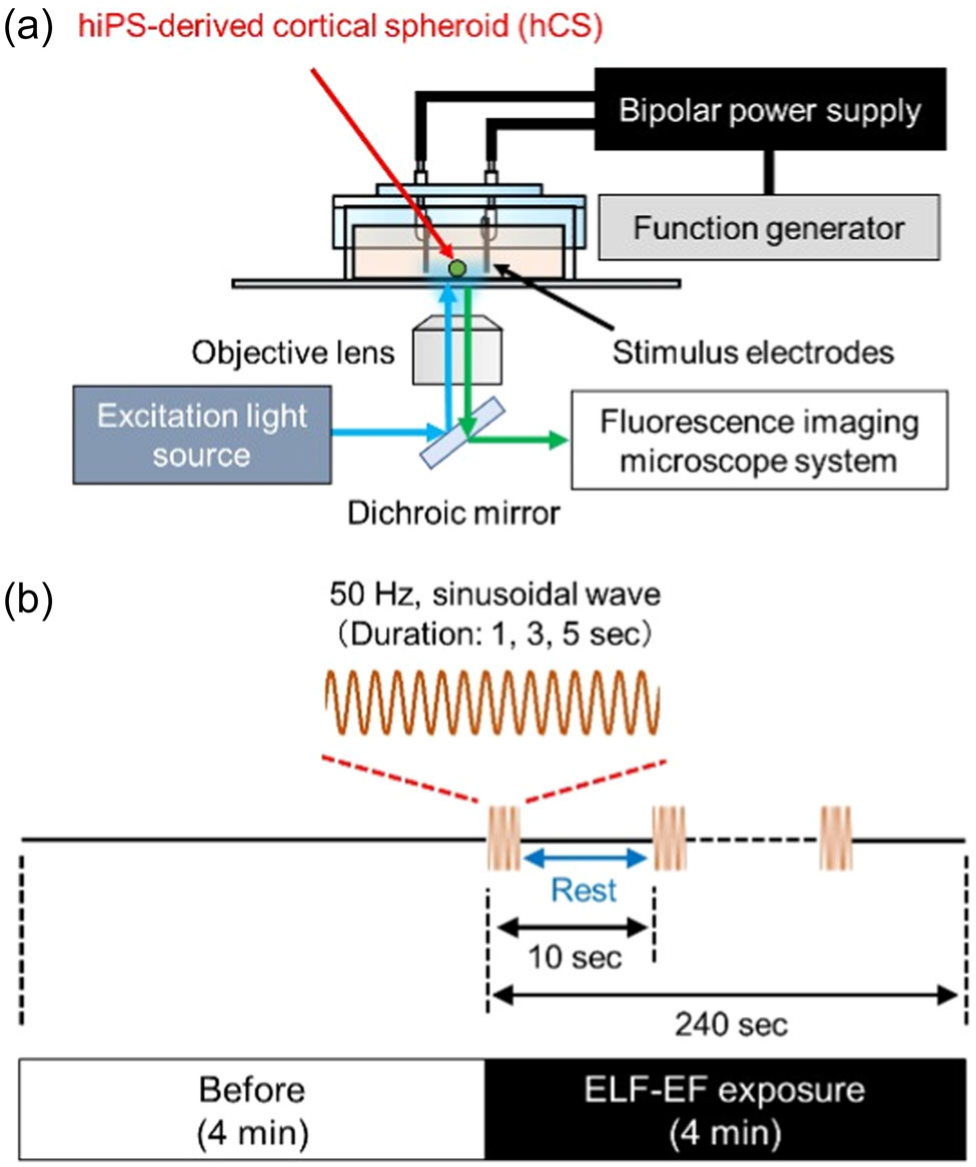
Experimental setup and extremely low‐frequency electric field (ELF‐EF) exposure protocol. (a) ELF‐EF exposure and fluorescent calcium imaging setup for human induced pluripotent stem cell‐derived cortical spheroid (hCS). (b) ELF‐EF exposure protocol.

ELF‐EF exposure was performed using a specific exposure apparatus consisting of a parallel‐plate electrode coupled to a cell culture dish (Figure 1a). A high‐speed bipolar power supply (HSA4051; NF Corporation) was combined with the parallel‐plate electrode, and 50 Hz sinusoidal wave ELF‐EFs with a constant current‐controlled output applied via a function generator (WF 1974; NF Corporation). The size of the plate electrode was 20 × 10 mm, and the distance between the two electrodes set to 6 mm to ensure uniformity of the iEF distribution at the cell placement points based on the numerical dosimetry results. During the exposure experiment, hCSs were placed in the middle between the two electrodes and exposed to an ELF‐EF while [Ca^2+^]_*i*_ imaging was performed to examine the effects on [Ca^2+^]_*i*_ oscillation. The protocol used for ELF‐EF exposure is shown in Figure 1b. To evaluate changes in [Ca^2+^]_*i*_ oscillations before, during, and after exposure, each recording time was set to 4 min in each exposure experiment. During the 4‐min period, 24 exposures were performed, with the interval between the onset of successive exposures set at 10 s, whereas the duration of each exposure itself was 1, 3, and 5 s in a repeating cycle. The exposure condition was established prior to performing the exposure experiments by evaluating the temperature increase during ELF‐EF exposure. A fiber‐optic thermometer (FL‐2000; Anritsu) was used to confirm the temperature increase in the culture medium during ELF‐EF exposure and fluorescence imaging. By evaluating the temperature change before, during, and after exposure, we set the input voltage such that the temperature increase did not exceed 37 ± 0.2°C at the maximum exposure duration (5 s and 24 exposures).

### 2.5 Numerical dosimetry

**Figure 2:**
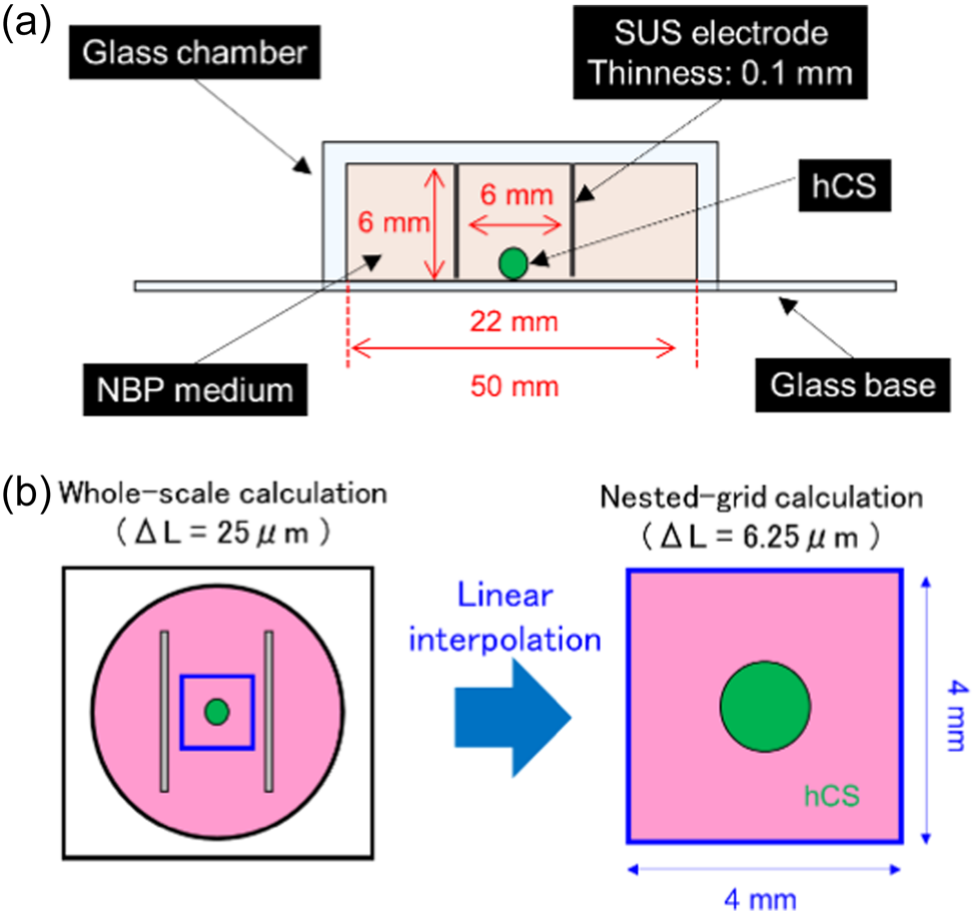
Voxel model used in numerical dosimetry for the induced electric field in human induced pluripotent stem cell‐derived cortical spheroids (hCS) during extremely low‐frequency electric field exposure. (a) Schematic view of the voxel model for numerical dosimetry. (b) Schematic view of the whole‐scale and nested‐grid calculation process.

Based on the configuration of the ELF‐EF exposure apparatus with parallel‐plate electrodes, as shown in Figure 2a, we constructed a numerical model. The hCS was placed between the two electrodes in the culture solution. Numerical dosimetry to calculate the iEF distribution in the hCS and culture solution was performed using a scalar potential finite difference calculation method (Dawson et al., 1996). The simulation was performed using a program code developed in‐house at the Central Research Institute of Electric Power Industry (Sekiba & Yamazaki, 2020). This method is widely used for numerical dosimetry in the human body exposed to electromagnetic fields at low frequencies (<100 kHz) (Dawson et al., 1996). For the conductivity of hCS, we used 0.1 S/m, which is the conductivity of gray matter at 50 Hz (Dimbylow, 2005). Although the relationship between conductivity of the hCS and human gray matter has not been compared based on measurements, it is assumed that the hCS contains similar dense aggregations of neuronal cell bodies as those in human brain gray matter. In addition, as shown below, we applied a conductivity similar to that of neuronal cell bodies in modeling the hCS, as one cell is represented by one voxel. The boundary between the culture solution and glass chamber/glass bottom was assumed to be isolated, where the current density in the normal direction is zero.

Similar to the method described by Dimbylow (2000), the numerical method used comprised a two‐step process involving full‐scale and nested‐grid calculations to account for the size difference between the parallel plate electrode and hCS, as shown in Figure 2b. In the first step, the calculations for the whole system model, including the culture solution and electrodes (without the hCS), were performed with a spatial resolution of 25 μm and number of cell diameter‐directed subdivisions of 40. Then, in the second step, the vicinity of the tissue (*X*, *Y*: ±2 mm, *Z*: 0–2 mm) was cut out and a nested‐grid calculation performed with a fourfold increase in resolution to Δ*L* = 6.25 μm (number of cell diameter‐directed divisions: 160). The electric potential values for the outer boundary in the nested‐grid calculations were linearly interpolated from results of the whole‐system calculations, and the isolated boundary condition applied without extrapolation only to the bottom surface. In addition, to determine the location of the hCS, we estimated the iEF distribution without the hCS using a full system calculation. Table 1 lists the parameters used in the numerical calculations. The electrical conductivity of 1.76 S/m reflects the measured data of the culture solution used in our experiments. To evaluate the maximum value in the hCS, the 99.9th percentile value of the hCS voxels was calculated. The 99.9th percentile has been adopted as a metric in previous studies to avoid unnecessary underestimation in the case of nonuniform exposure (Shiina et al., 2021).

**Table 1:** Numerical calculation parameters.

Parameters	Set values
Frequency	50 Hz
Conductivity	NBP Medium: 1.76 S/m hCS: 0.1 S/m
Applied voltage	3 Vpp (electrode‐to‐electrode voltage, ±1.5 Vpp)
Voxel size	Whole‐scale calculation: 25 μm Nested‐grid calculation: 6.25 μm
Total number of voxels (nodes)	Whole‐scale calculation: 144,615,360Nested‐grid calculation: 2,144,432
Determination for linear simultaneous equations	Relative error: |*b*‐Ax | /|*b* | <1*E*−8

## 3 RESULTS

### 3.1 hCS functional evaluation

Changes in spontaneous electrical activity are associated with the development of functional neuronal networks. Therefore, we evaluated the pattern of spontaneous electrical activity during the development process of hCSs in the ELF‐EF exposure experiments. In this study, hCSs with a diameter of approximately 1 mm, which is the same size used in numerical dosimetry, were selected from 96‐well plates and subsequently placed onto 64‐electrode MEA electrode arrays to evaluate changes in spontaneous electrical activity for up to 60 days using the MEA system. The MEA system is noninvasive to cultured cells; therefore, long‐term assessment of neuronal activity in the same hCS was possible.

**Figure 3:**
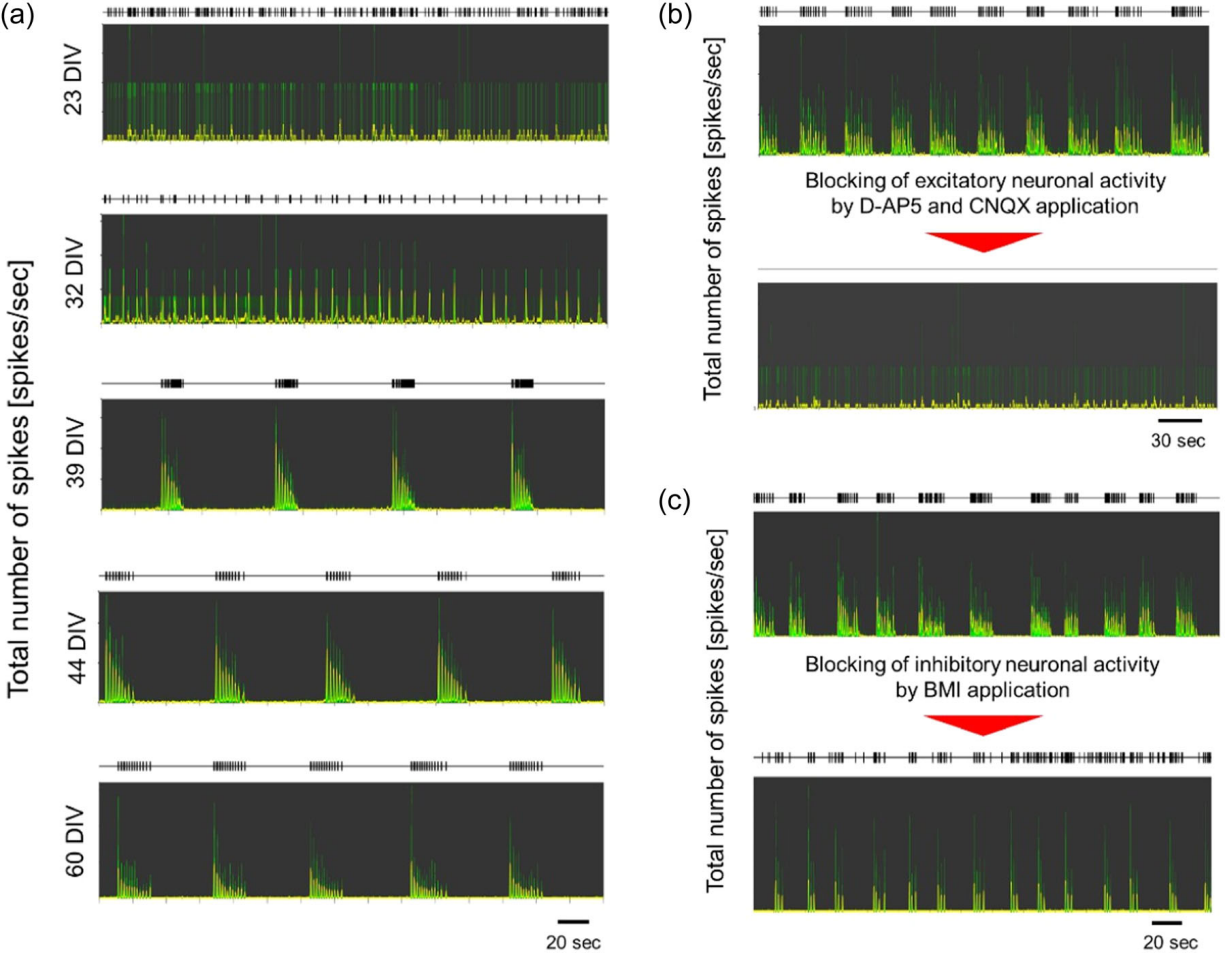
Firing pattern of the functional neuronal network during the developmental process of human induced pluripotent stem cell‐derived cortical spheroids using the multielectrode array recording system. (a) Changes in firing pattern during the developmental process of the functional neuronal network at 23, 32, 39, 44, and 60 days in vitro (DIV). The black trace in each figure represents the time stamp of spikes in a synchronized bursting activity. (b) Disappearance of synchronized bursting activity after the addition of D‐(‐)‐2‐amino‐5‐phosphonopentanoic acid (D‐AP5) and 6‐cyano‐7‐nitroquinoxaline‐2,3‐dione (CNQX). (c) Frequency association of synchronized bursting activity after the addition of 1(*S*),9(*R*)‐(‐)‐bicuculline methiodide (BMI).

Changes in the firing pattern of neuronal activity during the development of functional neuronal networks are shown in Figure 3a. Recording data at 23 and 32 days in culture showed a high density of spikes and no regularity in firing patterns. In contrast, synchronized bursting activity occurred after 39 days in culture, and these bursts persisted until approximately 60 days. In addition, the time interval of synchronized bursting activity was stable. Based on these results, we decided to use the hCS for ELF‐EF exposure experiments in this study, under conditions where [Ca^2+^]_*i*_ bursts were stabilized by long‐term incubation for approximately 1.5–2 months. In addition, hCSs cultured for approximately 2 months were evaluated with antagonists in excitatory and inhibitory neurons.

Figure 3b shows that the addition of D‐AP5 and CNQX resulted in the disappearance of synchronized bursting activity, confirming the presence of excitatory neurons within the hCS. Figure 3c shows that the number of synchronized burst activities increased after the addition of BMI, suggesting an association with increased excitatory neuronal activity and confirming the presence of inhibitory neurons. These results indicate that the hCS used in the ELF‐EF exposure experiments likely contained excitatory and inhibitory neurons that constitute the human cortex‐derived neuronal network of the cerebral cortex.

### 3.2 Modulation of [Ca^2+^]_*i*_ oscillation during ELF‐EF exposure

**Figure 4:**
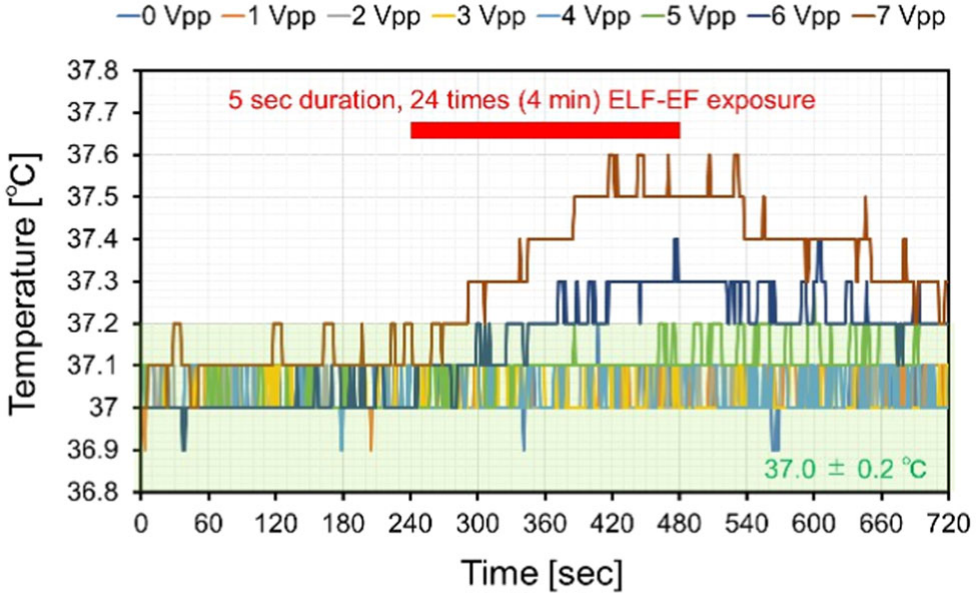
Temperature of the culture medium during extremely low‐frequency electric field (ELF‐EF) exposure at each voltage input.

Before ELF‐EF exposure to hCS, we evaluated temperature changes in the culture solution during repeated exposure. Temperature changes were recorded for 4 min before and after field exposure using a fiber‐optic thermometer. The results of the temperature evaluation are shown in Figure 4. The results showed that the input voltage at 0–4 Vpp was within 37.0 ± 0.1°C, and that at 5 Vpp was within 37.0 ± 0.2°C, when compared with the set temperature of 37.0°C. However, at ≥6 Vpp, the temperature rose well above 37.0 ± 0.2°C. Based on these results, we performed ELF‐EF exposure experiments under conditions where the upper limit of the input voltage was set at 5 Vpp.

**Figure 5:**
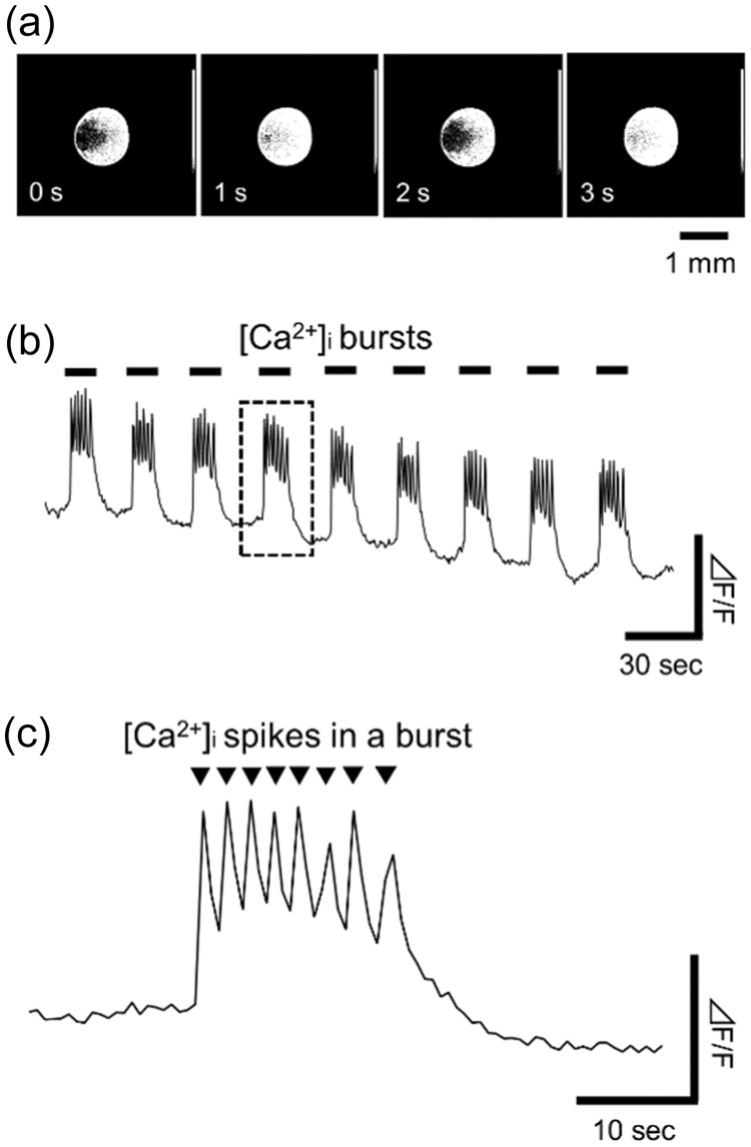
Wave form of spontaneous [Ca^2+^]_*i*_ bursts and spikes of human induced pluripotent stem cell‐ derived cortical spheroids. (a) Example of a fluorescent image showing periodic [Ca^2+^]_*i*_ activity. (b) Example of time‐series data demonstrating fluorescent changes of [Ca^2+^]_*i*_ bursts. (c) Example of time‐series data showing [Ca^2+^]_*i*_ spikes in a single [Ca^2+^]_*i*_ burst.

During the exposure experiments, hCSs that had been incubated for more than 2 months and produced synchronized bursting activity were placed between parallel‐plate electrodes to study the effects of ELF‐EF exposure on [Ca^2+^]_*i*_ oscillation. The samples used in the exposure experiments were selected after confirming the size of the hCS with a microscope before the experiment, and those that fell within the range of 0.9–1.1 mm in diameter were selected. The recording of spontaneous neuronal network activity using fluorescence imaging revealed a firing pattern of [Ca^2+^]_*i*_ oscillations, as shown in Figure 5a,b. The obtained fluorescence data of [Ca^2+^]_*i*_ oscillations had characteristics similar to those of the synchronized bursting activity observed by extracellular recordings using the MEA system, as shown in Figure 3a. Therefore, the [Ca^2+^]_*i*_ oscillations obtained from hCSs reflected the pattern of electrical activity in the neuronal network. In addition, a single burst‐type [Ca^2+^]_*i*_ oscillation contained several spike‐type [Ca^2+^]_*i*_ oscillations, as shown in Figure 5c. Therefore, we investigated the effects of ELF‐EF exposure on two types of firing patterns: burst‐type [Ca^2+^]_*i*_ (defined as [Ca^2+^]_*i*_ bursts) and spike‐type [Ca^2+^]_*i*_ oscillations in a single [Ca^2+^]_*i*_ burst (defined as [Ca^2+^]_*i*_ spikes). As shown in Figure 5, the fluorescence intensity of the hCS was notably changed, and the [Ca^2+^]_*i*_ burst and spike activity could be clearly distinguished in all samples. Therefore, the frequency of these events was visually counted during the ELF‐EF exposure experiments.

**Figure 6:**
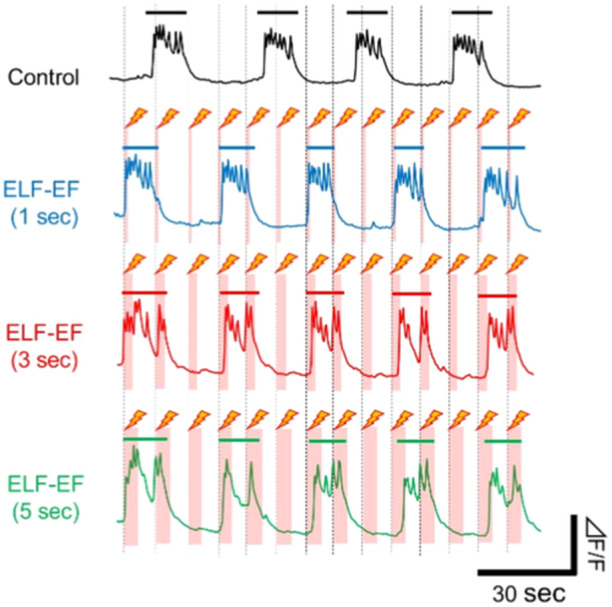
Stimulus effects of extremely low‐frequency electric field (ELF‐EF) exposure on the [Ca^2+^]_*i*_ oscillation in human induced pluripotent stem cell‐derived cortical spheroids with different durations (Input: 3 Vpp, Duration: 1, 3, and 5 s). Modulation of the waveform and number of [Ca^2+^]_*i*_ spikes during ELF‐EF exposure with different durations. The bars in each waveform indicate the [Ca^2+^]_*i*_ bursts.

Figure 6 shows an example of the change in [Ca^2+^]_*i*_ oscillation observed before and after the application of a 3 Vpp input voltage for different durations (1, 3, and 5 s). A clear change in the waveform of [Ca^2+^]_*i*_ oscillations during ELF‐EF exposure could be observed depending on the duration of application. These results indicate that the time interval during which [Ca^2+^]_*i*_ bursts occur tends to be shorter in the 3 Vpp exposure than in the absence of the same exposure for all time widths (1, 3, and 5 s). However, the stimulus effect on [Ca^2+^]_*i*_ spikes varied depending on the timing of the ELF‐EF exposure. ELF‐EF exposure increased the number of [Ca^2+^]_*i*_ bursts and spikes immediately before or during synchronized bursting activity, but these effects could not be confirmed immediately after the [Ca^2+^]_*i*_ burst. These results indicated that the engineered hCS exhibited stimulus responsiveness that was heterogeneous in terms of stimulus intensity and width. In addition, at an applied voltage of 3 Vpp, the effects on stimulus‐evoked responses and [Ca^2+^]_*i*_ oscillations were observed for periods of 1, 3, and 5 s. Therefore, we investigated the level of ELF‐EF exposure that would affect [Ca^2+^]_*i*_ oscillations, using 3 Vpp as the upper limit.

### 3.3 Effective values under ELF‐EF exposure on [Ca^2+^]_*i*_ oscillation in hCS

To evaluate the stimulus effects of ELF‐EF exposure on [Ca^2+^]_*i*_ oscillations in hCS, the number of [Ca^2+^]_*i*_ bursts and spikes within 4 min before and during exposure were compared.

**Figure 7:**
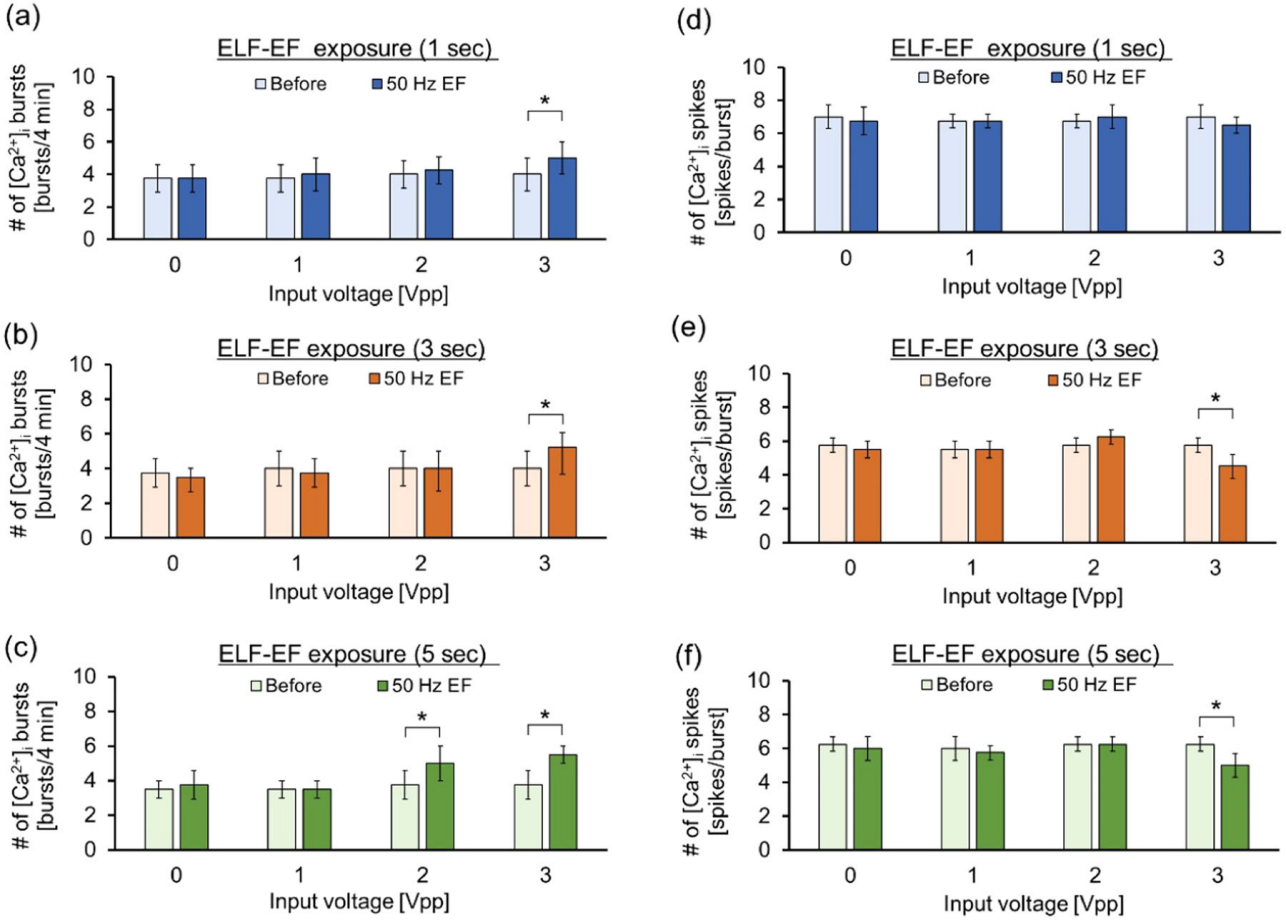
Stimulus effects of extremely low‐frequency electric field (ELF‐EF) exposure on [Ca^2+^]_*i*_ bursts and spikes. Effect of the number of [Ca^2+^]_*i*_ bursts during 0–3 Vpp inputs with durations of (a) 1 s, (b) 3 s, and (c) 5 s. *p < 0.05, *N* = 4. Effect of the number of [Ca^2+^]_*i*_ spikes during 0–3 Vpp inputs with durations of (e) 1 s, (f) 3 s, and (g) 5 s. *p < 0.05, *N* = 4.

Figure 7 shows the stimulus effects of ELF‐EF exposure on [Ca^2+^]_*i*_ oscillations. The frequency of the [Ca^2+^]_*i*_ burst was statistically increased during exposure for all time widths at an applied voltage of 3 Vpp (Figure 7a–c). An increase in the [Ca^2+^]_*i*_ burst frequency was observed when the exposure duration was 5 s, even at an applied voltage of 2 Vpp (Figure 7c). Therefore, the ELF‐EF exposure threshold for increasing [Ca^2+^]_*i*_ burst frequency was inversely related to the exposure duration. The relationship between the intensity and duration of ELF‐EF exposure on the frequency of [Ca^2+^]_*i*_ spikes is presented in Figure 7d–f. The results shown in Figure 7e,f indicate that the number of [Ca^2+^]_*i*_ spikes significantly decreased at 3 and 5 s of ELF‐EF exposure, whereas no decrease was observed at other exposure conditions.

### 3.4 Numerical estimation of iEF distribution in hCS under ELF‐EF exposure

**Figure 8:**
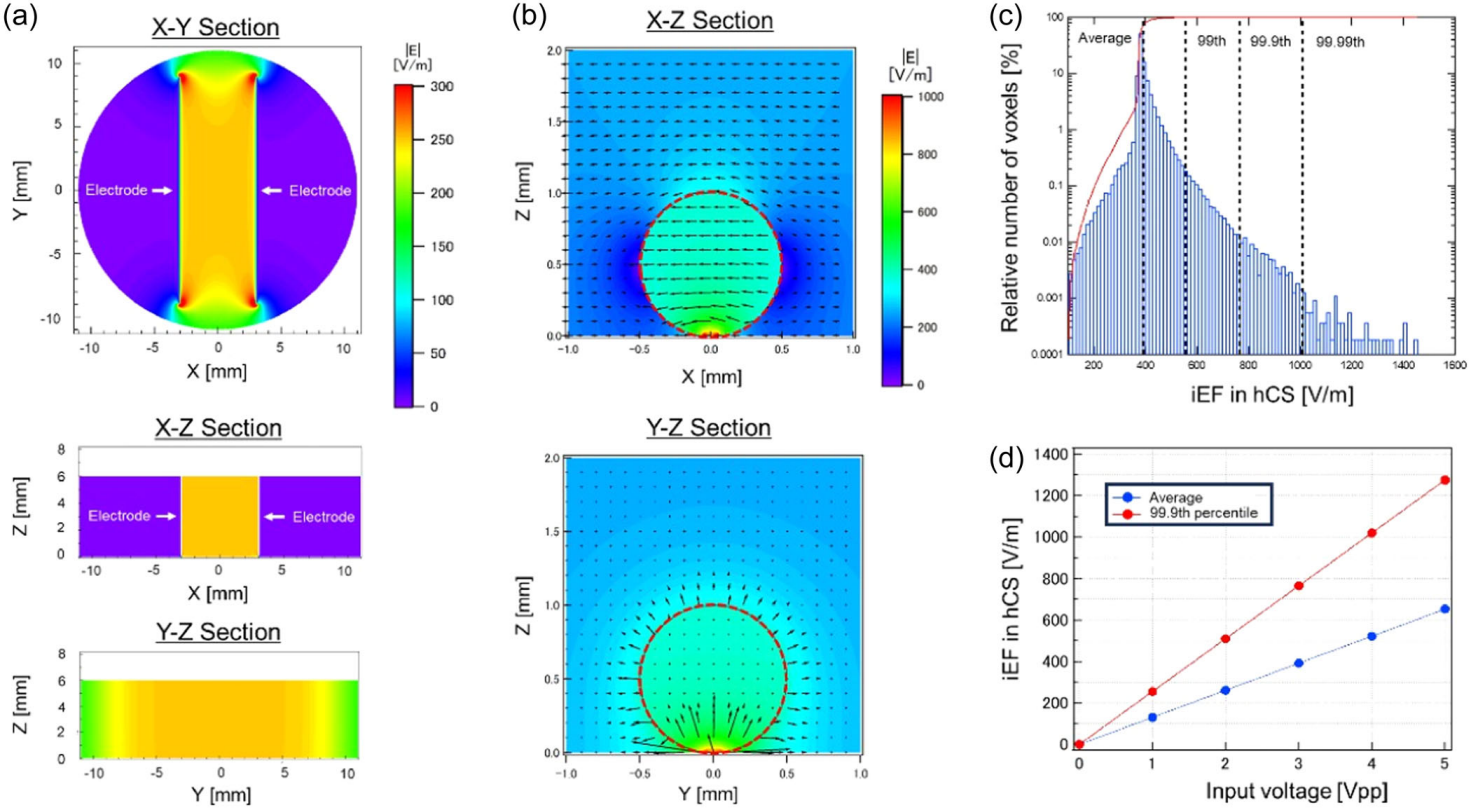
Numerical estimation of the induced electric field (iEF) in culture solution and human induced pluripotent stem cell‐derived cortical spheroids (hCS). (a) Distribution of the iEF in culture solution for the X–Y, X–Z, and Y–Z sections without hCS. (b) Distribution of the iEF in hCS for the X–Z and Y–Z sections. The red circle shows the hCS area. (c) Histogram of the relative number of voxels. (d) Relationship between the applied input voltage value and strength of the iEF in hCS (peak [99.9th percentile vs. average]).

To estimate the intensity of the iEF in the hCS under ELF‐EF exposure conditions, numerical dosimetry was performed for the model of the ELF‐EF exposure apparatus (Figure 8).

Figure 8a shows the calculated results of the iEF distributions for the X–Y and Y–Z cross‐sections at the electrode edge applied with a voltage of 3 Vpp under the condition without hCS. The iEF distribution for the X–Z section was generally uniform within a range of ±5 mm. However, the 100‐μm gap between the electrode and the bottom of the culture dish caused a complex change in the iEF distribution directly below the electrode. Therefore, when performing iEF exposure experiments, the hCS was placed within ±1 mm of the center of the culture dish.

Figure 8b shows the calculated results of the iEF distribution in the hCS for a nested grid model when the hCS was placed in the center of the culture dish. The calculated results in the X–Y, X–Z, and Y–Z cross‐sections indicated that a nonuniform iEF distribution was formed on the bottom of the hCS and in the surrounding area, and that the iEF in the hCS was higher than that in the culture solution. In particular, the iEF hotspot was found at the contact points between the bottom of the culture dish and the hCS. Therefore, we estimated the maximum value at the bottom and averaged the value within the hCS. Figure 8c shows the histogram of the relative number of voxels. Above the 99.9th percentile value, the number of relative voxels varied. From these results, we defined the 99.9th percentile value as a reasonable peak value in numerical dosimetry. The relationship between the applied voltage and iEF strength within the hCS is shown in Figure 8d. The iEF strength at each applied voltage value of 1–5 Vpp was estimated with average values ranging from 130.5–652.6 V/m and maximum values (99.9th percentile values) ranging from 254.9 to 1274.4 V/m. In this study, the average value of iEF intensity in the hCS at an applied voltage of 3 Vpp, which affected the frequency of [Ca^2+^]_*i*_ oscillations in the hCS, was 391.6 V/m (average). The 99.9th percentile value, which estimated the maximum exposure level of the iEF strength, was 764.6 V/m (peak). This indicated that there was approximately a twofold difference between the mean and 99.9th percentile values.

## 4 DISCUSSION

The hCSs, differentiated from hiPSC‐derived cortical neurons with a 3D structure, were used to evaluate the stimulus effects of ELF‐EF exposure on [Ca^2+^]_*i*_ oscillation. In previous studies (Hörberg et al., 2022; Trujillo et al., 2019) using MEA to evaluate electrical activity during the development of hCS or human brain organoids, periodic synchronized bursting activity was observed under long‐term culture. In our hCS samples, synchronized bursting patterns were first observed 5–6 weeks after induction and stabilized after 7–9 weeks. The spontaneous activity patterns in our experiments were similar to those of a previous study (Hörberg et al., 2022) that used hiPSC‐derived neural spheroids. However, the timing of the generation of synchronized bursting activity varies from sample to sample; therefore, it is necessary to examine the state of the hCS before performing exposure experiments.

Subsequently, we applied [Ca^2+^]_*i*_ imaging of hCSs after synchronized bursting activity was stabilized, and periodic [Ca^2+^]_*i*_ oscillations were observed, similar to the electrical activity recorded by the MEA system. The flux of [Ca^2+^]_*i*_ ions across the neuronal cell membrane is associated with the generation of action potentials; therefore, the [Ca^2+^]_*i*_ oscillation reflects the synchronized electrical activity of the neuronal network. The waveform of the [Ca^2+^]_*i*_ oscillation identified in our experiments was similar to that observed in a previous study (Woodruff et al., 2020) when cyclothiazide (CTZ) was added to hCSs. CTZ, a positive allosteric regulator of AMPA receptors, has been associated with seizure‐like neuronal activity (Qi et al., 2006). Therefore, our results suggest that the AMPA receptors are strongly involved in the neuronal activity of hCSs.

In the exposure experiments, hCSs cultured for 2 months were used to evaluate the effect of ELF‐EF on the generation of spontaneous and synchronized bursts of [Ca^2+^]_*i*_ oscillations. Here, we use the synchronized bursting activity of the whole area of an hCS as an indicator in the ELF‐EF exposure experiments, and this activity also temporally coincided with the bursting activity measured via the MEA system. Thus, optical imaging of [Ca^2+^]_*i*_ oscillation has the advantage of providing a real‐time, low‐noise assessment of the stimulus responses of repetitive ELF‐EF exposure on the synchronized bursting activity of cultured neuronal networks. Our results showed that the frequency of [Ca^2+^]_*i*_ oscillations at both time intervals increased when an input voltage of 3 Vpp was applied. As shown in Figure 6, some [Ca^2+^]_*i*_ bursts coincided with the timing of electrical stimulation (e.g., the first ELF‐EF exposure). This effect suggests that, under certain conditions, it may serve as a trigger for the synchronized burst activity. In a previous study, electrical stimulation was shown to trigger synchronized bursting activity, and the mechanisms of this phenomenon may involve the presence of highly active neurons in the functional organization of the cortical microcircuit (Pasquale et al., 2017). Indeed, these neurons, called “major burst leaders,” have been identified by their leadership property, which is the origin of the generation of synchronized bursting activity (Eckmann et al., 2008; Ham et al., 2008). Therefore, we also considered that the acceleration of [Ca^2+^]_*i*_ bursts observed in our experiments was related to the effects of ELF‐EF exposure on major burst leader neurons. However, the effect of stimulation during [Ca^2+^]_*i*_ burst generation is to modulate the firing pattern of the synchronized bursting activities. Here, previous studies have indicated a mechanism of epileptiform‐activity suppression during 50 Hz EF stimulation that is related to an inhibition of neuronal activity by inducing potassium efflux and depolarization (Bikson et al., 1999, 2001; Durand & Bikson, 2001). The culture (rat hippocampal slices) and electrical stimulation methods used were different from those used in our study, but these results provide an important perspective on the mechanism by which bursts of neuronal activity are inhibited by electrical stimulation‐induced potassium efflux. In addition, there is a period immediately after the onset of a burst when no stimulating effect occurs, and this phenomenon may involve a refractory period in excitatory neurons. Our experimental results showed that the response to ELF‐EF exposure to an hCS composed of multiple functionally distinct excitatory and inhibitory neurons is complex. Therefore, the thresholds and mechanisms of stimulus‐response should be investigated in the future by using other hCS models that reflect the neuronal cell types associated with different cortical regions.

As a clear effect on [Ca^2+^]_*i*_ oscillations was observed at an applied voltage of 3 Vpp, the threshold of the iEF intensity required to modulate [Ca^2+^]_*i*_ oscillations *w* was estimated by decreasing the applied voltage in steps of 1 Vpp. The results showed that the minimum threshold for affecting the frequency of bursts of [Ca^2+^]_*i*_ oscillations was 2 Vpp under 5 s of ELF‐EF exposure. For the other conditions, no effects were observed below 3 Vpp. These results suggest that the minimum exposure level at which the effects occurred was 2 Vpp for the applied voltage under the experimental conditions, and that the stimulus‐response depends on the generation of spontaneous [Ca^2+^]_*i*_ oscillations and the timing of the application of ELF‐EF exposure.

Various studies have been conducted on the effects of exposure to EF and MF on changes in intracellular [Ca^2+^]_*i*_ concentrations (Golbach et al., 2016). The [Ca^2+^]_*i*_ oscillations play an important role in intracellular signaling (Berridge et al., 2003) and are observed in neuronal cells as short‐term activity with periodicity associated with neuronal electrical excitability. The results of these studies confirmed that the percentage and amplitude of cells with [Ca^2+^]_*i*_ concentrations did not change during or after exposure to relatively low‐intensity EFs; however, the frequency of oscillations increased significantly (Golbach et al., 2016). The increase in the frequency of [Ca^2+^]_*i*_ oscillations reported in our study suggests that we had observed the same phenomenon as that in previous studies. However, our experiments used a high‐intensity ELF‐EF, which is different from the mechanism of [Ca^2+^]_*i*_ influx caused by exposure to a relatively weak MF, as reported in a previous study (Golbach et al., 2016). In contrast, changes in [Ca^2+^]_*i*_ oscillations are also involved as second messengers in the activation of downstream proteins (Smedler & Uhlen, 2014) and can, therefore, potentially modulate cell behavior. In this study, we aimed to estimate the ELF‐EF exposure levels that require [Ca^2+^]_*i*_ oscillation modulations; therefore, we did not evaluate the functional effects on neuronal cells. Our experimental results suggest that the stimulus effects reflect electrical excitation of the cell membrane.

In this study, we estimated the distribution of an iEF within an hCS using nested‐grid‐based numerical dosimetry. Recently, the numerical dosimetry of EF and MF exposure to the human brain has been accurately evaluated using large‐scale human body models (Alonso et al., 2023; Diao et al., 2023; Gomez‐Tames et al., 2020; Hirata et al., 2011, 2021; Makarov et al., 2017; Rashed et al., 2019; Soldati et al., 2020; Yokota et al., 2019). In contrast, most numerical dosimetry for cell experiments has been performed on cultured cells adhering to the bottom of culture dishes, and although certain results consider culture solutions (Consales et al., 2021) and even the surface of spheroids (Ye et al., 2021), the distribution of iEFs within hCSs has not been comprehensively elucidated. To address this issue, we estimated the iEF distribution within an hCS using nested‐grid calculations. The results of numerical dosimetry showed a local increase in iEF strength at the contact points between the culture dish and the hCS, as well as a large variation in the distribution of the iEF due to the current path. Therefore, when performing EF and MF exposure experiments using 3D‐cultured tissues, such as spheroids and organoids, evaluations based on numerical dosimetry must be performed. In particular, estimation of the concentration of iEFs and their variation is critical.

To compare our experimental results with theoretical neurostimulation thresholds described in international guidelines or standards, we referred to the IEEE standard C95.1‐2019 (IEEE, 2019). In our experiments, the minimum applied voltage value at which changes in the number of burst‐type [Ca^2+^]_*i*_ oscillations were observed was 2 Vpp, and the iEF strength estimated to be 510 V/m (peak) and 261 V/m (average) at 2 Vpp. In addition, the effect on the number of [Ca^2+^]_*i*_ spikes in a single [Ca^2+^]_*i*_ burst could only be observed at 3 Vpp. These results indicate that the iEF strength at which changes in spike‐type [Ca^2+^]_*i*_ oscillations occurred was above approximately 765 V/m (peak) and 392 V/m (average) at 3 Vpp. Here, the theoretical threshold for 10‐μm myelinated nerve fibers in the central nervous system at 50 Hz was 12.3 V/m (peak), which is described in the IEEE standard (IEEE, 2019). Therefore, our experimental results reported values at least more than 20 times higher than the theoretical threshold of a 10‐µm myelinated nerve fiber stimulation (IEEE, 2019; Reilly, 1998). Based on these findings, the results of our experiment suggest that the [Ca^2+^]_*i*_ oscillations of hCSs have a certain robustness as a neuronal response to ELF‐EF exposure, or the diameter of nerve fibers contained in hCSs was less than 1 μm. In fact, the thickness of nerve fibers distributed in the human brain and central nervous system ranged from 0.16 to 9 μm, with 0.6 μm being particularly common, as reported in a previous study (Liewald et al., 2014). The environments for the human body and cultured neurons are very different. The hCS used in this experiment nerve fibers with uncertain thicknesses, but the nerve stimulation thresholds (not synaptic effects) of 3D and spontaneously oscillating neuronal networks may be more robust to ELF‐EF exposure than the thin nerve fibers in a single neuron are. To address this issue, it would be useful to investigate the relationship between detailed assessment of the thickness of nerve fibers, such as axons or dendrites, in different types of neuronal cells in the central nervous system or in the hCS and the stimulation threshold.

To reveal the stimulus thresholds of EF exposure on the neuronal population in an active state, we evaluated [Ca^2+^]_*i*_ oscillations associated with the synchronized electrical activity of the hCS during high‐intensity ELF‐EF exposure. The strength of the iEFs that caused changes in [Ca^2+^]_*i*_ oscillations of the hCS exceeded the excitation threshold of the neuronal cell membrane, as estimated from theoretical models. This suggests that the experimentally observed effects on neuronal network activity occurred via excitation of neuronal cell membranes and voltage‐dependent calcium channels.

Therefore, our results suggest that the excitation threshold of the hCS is more robust to external ELF‐ EF exposure than that of a thin nerve fiber (<1 μm in diameter) in a single neuron. In contrast, a previous study suggested that neuronal modulation by EF or MF exposure occurs below membrane excitation thresholds (Barnes & Greenebaum, 2020; Durand, 2003; Modolo et al., 2013a, 2013b; Saunders, 2003). In particular, several human studies have accurately assessed the threshold of neuronal modulation in human subjects (Bouisset et al., 2022; Davarpanah et al., 2017; Evans et al., 2019; Legros et al., 2012, 2015; Marino et al., 2004; Modolo et al., 2017). The hCS used in this experiment can also be applied to ELF‐MF exposure experiments, as in our previous experiments (Saito, Takahashi, et al., 2018; Takahashi et al., 2017, 2022, 2023). Therefore, conducting ELF‐MF exposure experiments using human neural spheroids or organoids, which are simplified model tissues of the human brain, could be useful in examining the threshold of neuronal network activity or [Ca^2+^]_*i*_ oscillations caused by ELF‐MF exposure as biological findings that contribute to the development of safety guidelines from a mechanistic perspective.

## 5 CONCLUSION

We used hCSs to evaluate the stimulus effects of ELF‐EF exposure on [Ca^2+^]_*i*_ oscillations accompanied by synchronized bursting activity. The response to ELF‐EF exposure depended on the timing of [Ca^2+^]_*i*_ oscillations, and the minimum threshold was distributed between 255–510 and 131–261 V/m for peak and average values, respectively. Numerical dosimetry indicated that the iEF strength inside the hCS had a complex distribution and was strong at the contact points to the culture dish. These findings suggest that the [Ca^2+^]_*i*_ oscillation of 3D neuronal networks is more robust to external ELF‐EF exposure than membrane excitation of a single neuron is.

## CONFLICT OF INTEREST STATEMENT

The authors declare no conflict of interest.

## ETHICS APPROVAL STATEMENT

The authors have nothing to report.

## Data Availability

The authors have nothing to report.
